# Cognitive Load and Foreign-Language Anxiety in Second-Language Learning: A Systematic Review and Meta-Analysis

**DOI:** 10.3390/jintelligence14090228

**Published:** 2026-09-21

**Authors:** Hong Yi, Wenqian Tang, Qiang Chen, Zhuo Wang

**Affiliations:** 1 College of Foreign Languages, Hubei Minzu University, Enshi 445000, China; 2 College of Foreign Languages, Hainan University, Haikou 570228, China; 3 Library of Yunnan University, Kunming 650091, China; 4 College of Educational Sciences, Qingdao University, Qingdao 266071, China

**Keywords:** cognitive load, foreign-language anxiety, second language learning, meta-analysis, task design, cognitive load theory, control-value theory

## Abstract

Cognitive load and foreign-language anxiety are often examined separately in second-language learning, although both may arise when limited processing resources are strained. This meta-analysis examined whether the two constructs covary and why their association matters for models of L2 performance. A PRISMA-guided review identified eleven eligible studies (*N* = 1250), which were synthesised using a random-effects model. Two independent machine coders repeated the full-text eligibility assessment, effect-size extraction, and quality appraisal; two of the authors then verified every coding against the source reports and resolved all discrepancies. This process recovered one wrongly excluded study and corrected one misextracted coefficient. Greater load was associated with greater anxiety, *r* = 0.41, 95% CI [0.29, 0.52], and the estimate was *r* = 0.37, 95% CI [0.27, 0.46], after the most influential study was removed. All included estimates were positive. A specification analysis that substituted every available alternative component, wave, subscale, subgroup, and path yielded pooled estimates from 0.38 to 0.45; setting all eleven studies simultaneously to their least and most favourable alternatives widened the range to 0.29–0.52. Heterogeneity was high, *I*^2^ = 80.1%, and the 95% prediction interval [0.03, 0.69] extended almost to zero. The pooled estimate therefore represents the centre of a dispersed literature rather than an expected result for a new study. An exploratory contrast between real-time and self-paced tasks was not significant and was confounded with language skill. The review also identified a reporting gap: thirteen additional reports measured both constructs but provided no statistic linking them. Conventional publication-bias diagnostics cannot address this form of selective non-reporting, which means that the pooled estimate is best regarded as an upper bound. This first construct-specific synthesis of the load–anxiety association connects cognitive architecture and attentional control with research on L2 anxiety and indicates why instructional studies should assess cognitive and affective outcomes together. Because the evidence is concurrent, predominantly self-reported, and drawn almost entirely from Chinese-speaking settings, it cannot establish that either construct causes the other or that both reflect a single mechanism.

## 1. Introduction

Second-language learning provides a demanding test of human cognitive architecture. Learners must maintain unfamiliar phonological, lexical, and syntactic information while integrating it with prior knowledge, selecting responses, and monitoring performance. These operations depend on a working-memory system with sharply limited capacity (Baddeley, 2003; Sweller, 1988). When task demands absorb that capacity, fewer resources remain for constructing and automating schemas—the knowledge structures that allow multiple elements to be processed as a single unit (Sweller, 1994; Sweller et al., 2011). Cognitive load therefore matters not only as an index of momentary difficulty but also as a constraint on whether limited processing resources can support learning. It can be indexed through subjective ratings of mental effort or through behavioural and physiological measures (F. Paas et al., 2003).

Two influential research traditions have examined the consequences of this constrained architecture through complementary lenses. Cognitive load research estimates the processing cost imposed by a task, text, or interface. In L2 learning, that cost varies with task complexity (Robinson, 2001; Robinson & Gilabert, 2007), individual working-memory capacity (Cho, 2018; Jung, 2018), and presentation format, including segmentation, signalling, and split attention (Mayer, 2009; Moreno & Mayer, 2007). Load has accordingly become a central outcome in evaluations of instructional and technology-mediated design.

Research on foreign-language anxiety examines the affective experience that accompanies many of the same processing conditions. Scovel (1978) distinguished language-related apprehension from trait anxiety, and Horwitz et al. (1986) operationalised foreign-language anxiety as a situation-specific construct encompassing communication apprehension, fear of negative evaluation, and test anxiety in the language classroom. It is now one of the field’s most extensively studied emotions (Dewaele & MacIntyre, 2014; Horwitz, 2001), and meta-analytic evidence links it to poorer achievement (Botes et al., 2020; Teimouri et al., 2019; X. Zhang, 2019). Importantly for an intelligence-oriented account, anxiety is not only an emotional state: worry, self-monitoring, and threat-related attention may compete with task-relevant executive control during learning and performance (MacIntyre & Gardner, 1991, 1994).

These research traditions converge on an unresolved question: When an L2 task places greater demands on limited processing resources, do learners also experience greater anxiety? Few studies have analysed this relationship directly. Among the 29 reports carried forward for detailed construct-level assessment in the present review, eleven reported a statistic relating the constructs and thirteen measured both but reported no linking statistic; four measured only one construct on full-text inspection, and one paired an objective load index with a general rather than a language-specific anxiety measure. Substantial information about the connection between cognitive cost and language-related anxiety has therefore remained unexamined.

This question matters for three reasons. First, attentional control theory proposes that anxiety can consume executive resources through worry and threat monitoring (Eysenck & Calvo, 1992; Eysenck et al., 2007), whereas control-value theory proposes that high demands combined with low perceived control can elicit anxiety (Pekrun, 2006; Pekrun et al., 2011). The theories differ in causal direction but make the same concurrent prediction: load and anxiety should be positively related. Second, a pooled estimate can show how large and how variable that relationship is across L2 settings. Third, if the outcomes reliably move together, evaluations of cognitively efficient instruction should also assess its affective consequences. Meta-analysis can answer whether the relationship is present and how strong it is; it cannot, by itself, explain why it occurs.

No single study provides a stable answer. Reported load–anxiety correlations range from negligible values to values above 0.70 across small samples, different language skills, and non-equivalent measures. The closest synthesis, by Gullo et al. (2025), provides an important point of comparison, developed in Section 5.3, but addresses a different question. That review combined cognitive load with working memory, attention, and need for cognition in a 13-study omnibus category. These constructs have different meanings and opposite functional polarity—greater working-memory capacity indicates more cognitive resources, whereas greater load indicates higher processing cost. A construct-specific meta-analysis is therefore needed to estimate both the average load–anxiety association and its between-study variation (Z. Chen et al., 2025).

Accordingly, the present study asks two questions: How strongly are cognitive load and foreign-language anxiety associated in L2 learners, and does the strength of that association vary with the pacing of the language task? The available evidence, however, places firm limits on the answers. It is predominantly concurrent, so the synthesis cannot determine whether load precedes anxiety, anxiety precedes load, or both depend on a third variable such as proficiency. Ten of the eleven pooled studies measured both constructs by self-report, making shared method variance a serious alternative explanation. The evidence base is also geographically narrow: ten of the eleven studies were conducted in Chinese-speaking settings—the Chinese mainland, Taiwan, and Hong Kong—so the estimate reported below describes learners in those settings far better than it describes L2 learners in general, a constraint returned to in Section 4.1 and Section 5.6. Within those limits, the study provides a first construct-specific estimate and identifies the measurement and research designs needed to test possible mechanisms.

## 2. Theoretical Background and Hypotheses

### 2.1. Cognitive Load in Second-Language Learning

Throughout this article, cognitive load denotes the momentary demand that a task imposes on working memory during performance, as experienced or exhibited by the learner. It is not used interchangeably with three neighbouring notions that recur in this literature and are kept distinct here: working-memory capacity, which is a comparatively stable individual-difference trait rather than a momentary state; task demand or task complexity, which is a property of the material or design rather than of the learner; and mental effort, which is the learner’s investment of resources and is one of the ways load is indexed rather than a synonym for it. Load is commonly differentiated into intrinsic load arising from the element interactivity of the material, extraneous load arising from presentation or task design, and germane processing directed toward schema construction and automation (Sweller, 2010). L2 tasks can tax the phonological loop through the maintenance of unfamiliar forms and the central executive through the coordination of form, meaning, response selection, and monitoring (Baddeley, 2003). Because working-memory capacity differs across learners, an identical task may impose different effective demands—an individual-differences issue that connects cognitive load directly to intelligence-related processing.

Load is measured either subjectively, through ratings of mental effort or differentiated load scales (Leppink et al., 2013; F. G. W. C. Paas, 1992; F. Paas et al., 2003), or through behavioural and physiological indicators such as secondary-task performance, response latency, pupillometry, eye movements, and processing-complexity proxies. In the present literature, syntactic dependency distance has been used as an objective proxy on the rationale that longer dependencies impose greater integration demands (Yan & Liang, 2022). Technology-mediated settings are especially relevant because interface and multimedia design can alter both the processing demands learners face and the emotions accompanying those demands (Mayer, 2009; Moreno & Mayer, 2007).

Measurement modality is consequential for interpreting a load–anxiety correlation. Subjective ratings are economical and sensitive, but they are retrospective and may be influenced by affect: an anxious learner may report greater effort partly because anxiety itself feels effortful. Objective indicators reduce this particular mono-method concern but capture only selected components of processing cost and are uncommon in classroom studies (F. Paas et al., 2003). The evidence is highly imbalanced across these modalities: ten pooled studies used subjective load measures, whereas only one used an objective index. The present synthesis can therefore describe, but not meaningfully compare, measurement modalities.

### 2.2. Foreign-Language Anxiety

Foreign-language anxiety is a situation-specific form of anxiety encompassing communication apprehension, fear of negative evaluation, and test anxiety in language-learning contexts (Horwitz et al., 1986; Horwitz, 2001). MacIntyre and Gardner (1991, 1994) showed that its implications extend beyond subjective discomfort: anxious learners may encode, process, and retrieve L2 material less efficiently. The construct is assessed globally with measures such as the Foreign Language Classroom Anxiety Scale and through skill-specific instruments for writing, reading, listening, speaking, and interpreting. This measurement diversity matters because a global classroom disposition and anxiety during a particular processing episode are not interchangeable.

The wider literature has examined foreign-language anxiety in relation to corrective feedback (Rassaei, 2015), sociobiographical characteristics (Y. Jiang & Dewaele, 2020), translanguaging and emotional safety (Dryden et al., 2021), and emotion regulation in technology-mediated learning (Z. Zhang et al., 2021). Two features of this literature are especially relevant to the present question. First, situation-specific anxiety should be more closely tied to current task demands than trait anxiety. Second, tasks differ in the temporal and evaluative control they afford: writing is typically self-paced and revisable, whereas speaking and consecutive interpreting often require public, time-constrained, and difficult-to-revise responses. These contrasts motivate a test of whether pacing conditions the strength of the load–anxiety association without presuming that pacing identifies its mechanism.

### 2.3. The Cognitive Load–Anxiety Nexus

Two accounts predict a positive association between cognitive load and foreign-language anxiety, but they assign different causal priority. From a control-value perspective, demanding tasks may reduce perceived control and thereby increase anxiety, particularly when learners judge their resources to be insufficient (Pekrun, 2006; Pekrun et al., 2011). From processing-efficiency and attentional control perspectives, worry and threat monitoring may occupy executive resources, requiring anxious learners to invest more effort to maintain performance (Eysenck & Calvo, 1992; Eysenck et al., 2007). In L2 learning, either process could operate, and reciprocal influence is also plausible. All three possibilities predict positive concurrent covariation.

This convergence is theoretically informative but inferentially limited. A positive correlation would be consistent with competition for limited executive resources and with demand-related appraisals of low control, yet it would not demonstrate either process. The same pattern could arise because proficiency, task difficulty, or response style affects both measures. The purpose of the synthesis is therefore to estimate the association and its dispersion, not to treat correlation as evidence of a shared mechanism. That distinction is especially important when relating the findings to intelligence research, where working-memory capacity and executive control are explanatory constructs rather than labels for any observed covariance.

At the level of testable predictions, anxiety-related worry and self-monitoring may function as task-irrelevant processing that consumes capacity, while intrinsically complex or poorly designed tasks may leave fewer resources available for emotion regulation. Both possibilities predict that greater load will co-occur with greater anxiety. A pooled estimate can quantify this relationship across the small, heterogeneous samples currently available and provide a basis for designing longitudinal or experimental studies capable of testing temporal direction.

**Hypothesis 1.**  *Cognitive load and anxiety are positively associated in L2 learners.*

### 2.4. Potential Moderators

Hypothesis 1 concerns the average association. A second, exploratory question concerns a possible boundary condition. Because meta-regression generally requires approximately ten studies per covariate, the present pool supports at most one binary contrast, and any such contrast can only be interpreted descriptively rather than as a well-powered test (Deeks et al., 2019; Thompson & Higgins, 2002). The contrast of interest distinguishes real-time, difficult-to-revise tasks from self-paced, revisable ones. When learners can slow down or revise, limited capacity may be partly accommodated through additional time; under real-time demands, resource shortfalls and low control are expressed more immediately. Both attentional control and control-value accounts could therefore predict a stronger association in real-time tasks. Two features of the available evidence, however, limit what such a contrast can establish, and both are documented in Section 4.3: task pacing is not separable from language skill in this pool, and almost every anxiety instrument refers to the course rather than to the task whose pacing is being classified. The contrast is accordingly posed as an exploratory question rather than as a hypothesis. Measurement modality is likewise treated as exploratory because the number of objective load studies could not be known in advance.

Exploratory question: Is the load–anxiety association stronger for anxiety arising in real-time, irreversible language tasks than for anxiety arising in self-paced, revisable ones?

### 2.5. The Present Study

The empirical record spans L2 writing (Lee et al., 2022; H. Wang et al., 2024; Yao & Fan, 2025), interpreting (Z. Jiang & Lv, 2026; Yan & Liang, 2022), listening (I.-J. Chen & Chang, 2009; Mekheimer, 2026), longitudinal classroom and gamified learning (Y. Chen et al., 2022; Hsu, 2017), and technology-mediated instruction (Peng et al., 2020; Puri et al., 2025). These studies use different instruments and report correlations or standardised paths. The present study places the eligible correlational estimates on a common scale to test Hypothesis 1 and then examines the task-pacing contrast as an exploratory question. Whether the association varies by load-measurement modality is also considered exploratory. Together, the analyses estimate the association, describe its limits, and specify what future studies must measure to test an intelligence-related processing account.

## 3. Method

### 3.1. Protocol and Reporting

**Figure 1:**
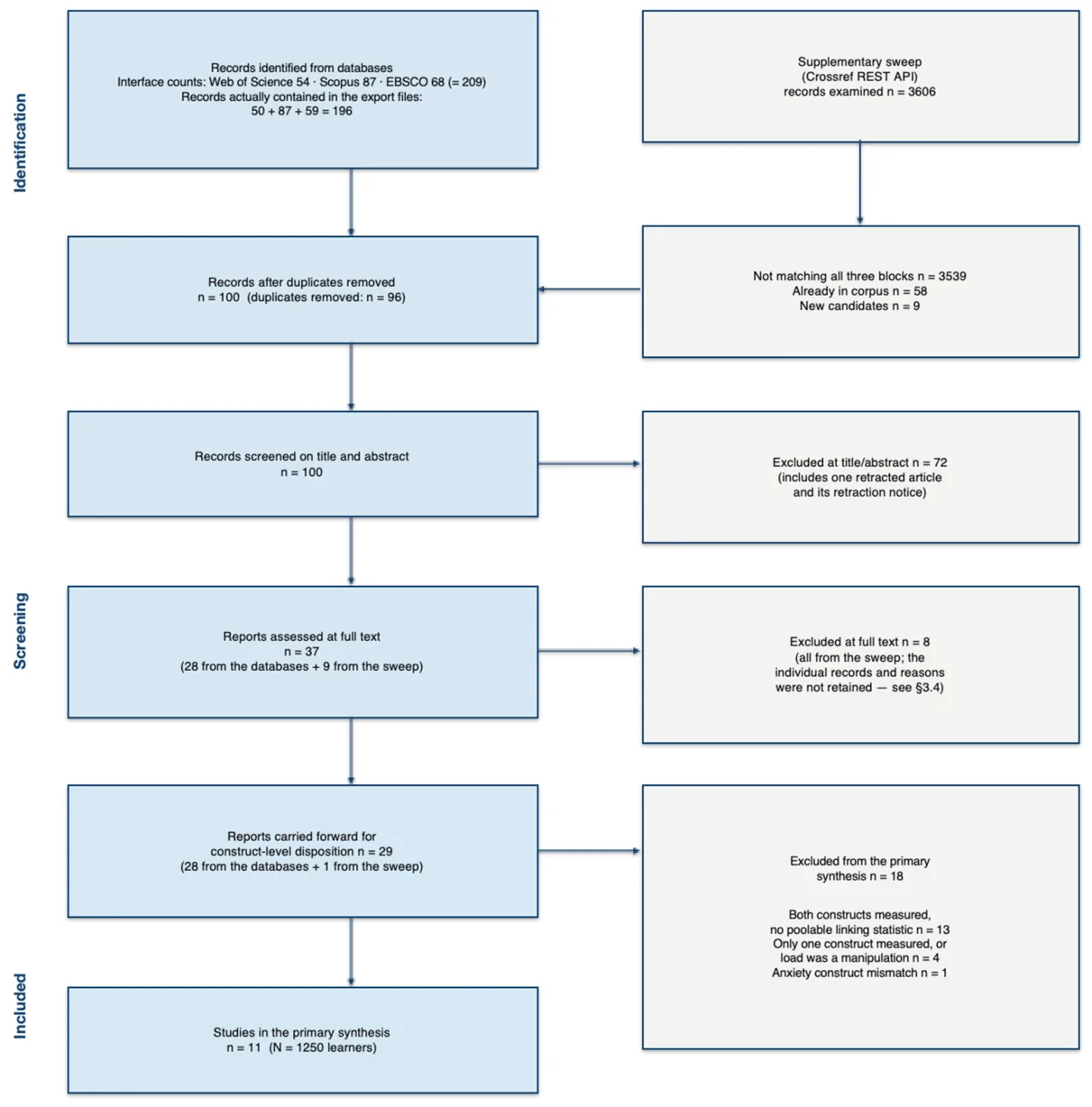
PRISMA 2020 flow diagram of study identification, screening, and inclusion.

The review followed the PRISMA 2020 statement (Page et al., 2021); a completed checklist is provided as Supplementary File S2, and the flow diagram appears as Figure 1. The review was not registered in PROSPERO or another prospective register, and no protocol was prepared or published in advance. The eligibility criteria, moderator coding, and analysis plan described below were fixed before the corresponding analyses were conducted but were not prospectively deposited; the implications are considered in Section 5.6. The review also followed established guidance for meta-analysis in the social sciences (Borenstein et al., 2009; Card, 2012; Lipsey & Wilson, 2001). Search yields, de-duplication, screening decisions, extraction, and analysis were recorded in a structured audit file. The first author initially conducted the screening, data extraction, and methodological appraisal. Two independent machine coders subsequently repeated these procedures, and two of the authors verified their codings against the source reports, as detailed in Section 3.5. The full screening record, analysed dataset, and analysis scripts are supplied as Supplementary Files S1–S4. No separate repository deposit was made; the Data Availability Statement refers only to these files.

### 3.2. Eligibility Criteria

Studies were eligible if they (a) reported primary empirical data; (b) sampled L2 or foreign-language learners of any target language or proficiency level; (c) measured cognitive load and anxiety specific to foreign- or second-language learning, including a particular L2 skill or task; and (d) reported, or allowed calculation of, an effect size relating the constructs. Cognitive load could be indexed by subjective load, mental effort or mental load, or an objective behavioural or physiological measure. General anxiety measures without an L2-specific component did not qualify for the primary synthesis; one report that paired an objective load index with the Beck Anxiety Inventory was excluded on this ground and reserved for a sensitivity model (Section 4.4). No restriction was placed on publication status. Journal articles, conference papers, dissertations, theses, and preprints were all eligible provided they met criteria (a) to (d); in this event, no dissertation, thesis, or preprint satisfied them, a limitation revisited in Section 5.6. Reviews, meta-analyses, retracted articles, conference abstracts, and records outside L2 learning were excluded, as were studies in which either construct appeared only as a theoretical frame or qualitative observation.

### 3.3. Information Sources and Search Strategy

The search combined three concept blocks—an affective block, a cognitive load block, and an L2-context block—across three platforms, and is reported here in enough detail to be re-run. On 1 June 2026, Web of Science Core Collection, Scopus, and EBSCOhost (ERIC, Academic Search Complete, PsycINFO, and Library, Information Science & Technology Abstracts) were searched without date or language restrictions; 1 June 2026 is the date on which each of these sources was last searched. The Web of Science query was TOPIC: anxiety AND TOPIC: (“cognitive load” OR “mental load” OR “mental effort” OR “load”) AND ABSTRACT: (L2 OR “second language” OR EFL OR ESL OR “foreign language” OR “language learn” OR “learn English” OR “English learn”); Scopus (TITLE-ABS-KEY) and EBSCO (ABSTRACT and SUBJECT) used the same Boolean logic. The stems “language learn”, “learn English”, and “English learn” were entered as open phrases so that they would also retrieve the inflected forms that carry most of this literature—language learning, language learner, learning English, and English learning—which a fully quoted phrase would have missed. The broad term “load” maximised recall but also retrieved irrelevant uses such as factor loadings and course load, which were removed during screening.

### 3.4. Selection Process

The searches returned 50, 87, and 59 records, respectively (*N* = 196). De-duplication by normalised DOI and title removed 96 records, leaving 100 for title-and-abstract screening. Of these, 72 were excluded, and 28 proceeded to full-text assessment. On 19 July 2026, a supplementary bibliographic sweep was run against the Crossref REST API (api.crossref.org/works), because the original EBSCO query had matched the L2 block against subject headings rather than abstracts, and the affective block had rested on the single term anxiety. Twenty query strings were submitted to the query.bibliographic field, which searches title, container title, author, and abstract metadata; each string paired one broadened affective term with one broadened load term, and results were filtered on the L2 block. The affective block was extended to apprehension, worry, and named instruments; the load block to cognitive demand, mental workload, and NASA-TLX; and the L2 block to interpreting, translation, and bilingualism. Returned records were de-duplicated against the existing corpus and among themselves on normalised DOI and title, and each remaining record was screened on its title and abstract against all three blocks. Of 3606 records examined, 3539 did not match all three blocks, 58 were already represented in the original corpus, and nine were new candidates. Full-text assessment of the nine excluded eight and retained one further study, I.-J. Chen and Chang (2009), an open-access article in a Spanish journal outside the original platform coverage. The audit trail for the supplementary sweep is incomplete: the eight reports excluded at that stage were not logged individually, so their identities and reasons for exclusion cannot now be reported. This is a departure from PRISMA 2020 item 16b and is recorded as such in the checklist; it affects only the supplementary sweep, and the record-level disposition of all 29 reports that reached construct-level assessment is complete and is given in Supplementary File S1. Backward citation searching of the reference lists of all included studies and of the adjacent synthesis by Gullo et al. (2025), together with forward citation searching of the same reports in Google Scholar on 19 July 2026, returned no additional eligible studies. In total, 37 reports underwent full-text assessment (28 from the database search and nine from the sweep), of which 29 were carried forward for detailed construct-level disposition. Eleven of the 29 entered the correlation meta-analysis. The remaining 18 did not: 13 measured both constructs but reported no statistic that could be pooled, four measured only one of the two constructs or treated load as an experimental manipulation rather than a measured score, and one paired an objective load index with a general rather than a language-specific anxiety inventory and was therefore reserved for the sensitivity analysis reported in Section 4.4. One further record identified at the title-and-abstract stage had been retracted and was excluded at that stage; it never entered full-text assessment.

### 3.5. Data Extraction and Coding

For each study, the first author extracted sample size, target language and skill, anxiety instrument and subtype, cognitive load instrument and modality, and the statistic relating load and anxiety. Because reproducing calculations from recorded inputs cannot establish that studies were correctly included or that coefficients were correctly located, the full-text stage was repeated in two additional passes. In the first, two independent machine coders re-made every eligibility judgement, every effect-size extraction, and every quality rating for all 29 reports. Each was a large-language-model agent supplied with the source reports and a written coding manual (Supplementary File S1) and with no access to the first author’s decisions or to the other agent’s output, and each was required to quote the sentence or table cell supporting every value it extracted. In the second pass, two of the authors independently checked each of those codings and each quoted piece of evidence against the source reports, and jointly settled every point on which the coders differed from one another or from the original extraction. Machine coding was used at the first of these stages because it makes an exhaustive, evidence-quoting re-reading of every report practicable, and human verification at the second because judgements of eligibility and measurement quality are not safely delegated. The agreement statistics below therefore describe the machine stage, and should be read with the caveat that two agents built on similar technology may share blind spots in a way that two independent human readers would not. Agreement between the two coders on whether a report entered the primary synthesis was complete (*κ* = 1.00); agreement between their consensus and the first author’s original disposition was *κ* = 0.854 (93.1%), with two discrepancies. On the finer three-way classification, agreement between the coders was *κ* = 0.895 (93.1%). Every discrepancy was adjudicated by the two verifying authors against the source reports, and each adjudication is documented with its reasoning in Supplementary File S1. More importantly, the procedure corrected two consequential errors. Mekheimer (2026) had been classified as reporting no poolable statistic because the focal analysis of cognitive load is a linear mixed model in which anxiety enters as an adjusted covariate; both coders located the zero-order Pearson correlations in the study’s covariate table, and the report was reclassified into the primary synthesis once the verifying authors had confirmed the values against the source. For Peng et al. (2020), the germane load coefficient (*r* = 0.319) had been extracted where the stated rule called for the intrinsic load coefficient (*r* = 0.194); both coders extracted the latter, and the value was corrected. Cognitive load modality was coded as subjective self-report or objective behavioural measurement; anxiety was coded by type, by language skill, and by whether the instrument referred to a specific task or to the course as a whole. To preserve effect-size independence, exactly one estimate was retained per study. The operative rule was to take the study’s most inclusive index of load, its total or overall score where one was reported, and to fall back on the intrinsic component only where components were reported without a total; where a study reported repeated measurements, the final concurrent wave was used. The wave rule was fixed in advance but was deliberately withheld from the coding manual, so that the coders’ independent choice would show whether it mattered; both selected the first wave, and that alternative is reported in the specification analysis. Both parts of the rule are conservative rather than favourable, since in Y. Chen et al. (2022), the only study to which the wave rule applies, the final wave yields the smallest of the five reported coefficients (*ρ* = 0.361 against 0.512, 0.540, 0.769, and 0.508). Alternative components and waves were retained and are reported as a full specification analysis in Section 4.4.

Methodological quality was appraised with the Mixed Methods Appraisal Tool (MMAT, 2018 version; Hong et al., 2018), using the five quantitative-descriptive criteria relevant to correlational evidence: sampling relevance, representativeness, measurement appropriateness, nonresponse bias, and statistical-analysis appropriateness. Each criterion was rated Yes, No, or Can’t tell against the full text by the first author and, independently, by the two machine coders, and every rating was then checked against the source reports by the two verifying authors. Agreement across the 55 study-by-criterion cells of the primary synthesis was 87.3%, or 48 of 55 cells, and the seven disagreements, six of them between Yes and Can’t tell, were settled by the verifying authors conservatively unless one appraisal cited specific evidence the others had not seen. Following MMAT guidance, criterion-level ratings are reported, and no summed quality score is computed, since the five criteria are not interchangeable and are not intended to be weighted equally. Studies were profiled rather than excluded on quality grounds, consistent with MMAT guidance and standards for L2 research reporting (Plonsky, 2013).

### 3.6. Effect-Size and Meta-Analytic Procedures

Correlations were transformed to Fisher’s z, with sampling variances calculated as 1/(*N* − 3), pooled with a random-effects model estimated by restricted maximum likelihood, and back-transformed to *r* (Borenstein et al., 2009; Hedges & Olkin, 1985). A random-effects model was specified a priori because languages, skills, instruments, and settings differed across studies. Heterogeneity was evaluated with Cochran’s Q, I-squared (Higgins et al., 2003), tau-squared, and a 95% prediction interval. Four inputs were not single zero-order Pearson coefficients and therefore required additional handling. For Puri et al. (2025), the source reports two standardised paths from cognitive load, one to speaking anxiety (*β* = 0.47) and one to anxiety about learning Chinese (*β* = 0.36). Each path comes from a simple bivariate regression with a single predictor, so the standardised coefficient is the zero-order correlation; this is confirmed by the reported coefficients of determination, since √0.22 = 0.469 ≈ 0.47 and √0.13 = 0.361 ≈ 0.36. No conversion formula was applied, and none is required. Because the two outcomes are anxiety measures obtained from the same 175 participants, they are not independent estimates and were averaged on the Fisher-z scale rather than entered separately, giving *r* = 0.417; each path is also reported singly in the specification analysis. For Y. Chen et al. (2022), the final-wave Spearman coefficient between foreign-language anxiety and intrinsic load was used (*ρ* = 0.361), with the other four waves reported in the specification analysis. For Hsu (2017), two independent subgroup Pearson correlations, *r* = 0.842 (*n* = 18, self-directed game) and *r* = 0.461 (*n* = 20, task-based game), were combined on the Fisher-z scale with inverse-variance weights, giving *r* = 0.686; the sampling variance of that combined estimate is 1/Σ(*n* − 3) = 1/32 rather than 1/(N − 3). The pairing of subgroup sizes to game versions follows the study’s Table 2, which reports them directly; Section 3 states only that one class of 20 was the experimental group and the other of 18 the control group, without saying which game each class used. For Mekheimer (2026), two Pearson correlations obtained from the same 60 participants under two counterbalanced within-participant listening conditions (*r* = 0.593 and *r* = 0.799) were averaged on the Fisher-*z* scale, giving *r* = 0.711, with the sampling variance based on the 60 participants rather than on 120 observations. One further input rests on a sample size the source does not state. Z. Jiang and Lv (2026) report their correlation matrix without giving the N behind it; the study has 39 participants who each completed three tasks, so the coefficient may rest on 39 learners or on 117 observations. We entered the participant-level figure, which is the conservative choice because it gives the study less weight, and the alternative changes nothing: entering 117 moves the pooled estimate from *r* = 0.411 to *r* = 0.417, which is *r* = 0.41 at the precision reported here. Because Hsu (2017) and Mekheimer (2026) combine Pearson coefficients, both belong with the zero-order inputs; the zero-order-only sensitivity model reported in Section 4.4 therefore retains them and excludes only the Spearman coefficient and the objective dependency-distance index. Small-study effects were examined with a funnel plot, Egger’s regression, rank correlation (Egger et al., 1997), and trim and fill (Duval & Tweedie, 2000), with the caution appropriate at *k* = 11. Robustness checks comprised leave-one-out analysis, sensitivity models restricted to zero-order correlations, models removing the objective load study and the study rated inadequate on statistical analysis, a model adding back the reserved construct-mismatch study, disaggregation of the subgroup study, and a full specification analysis substituting every alternative component, wave, subscale, subgroup, and path reported in the source studies. Mixed-effects meta-regression tested one binary contrast at a time (Deeks et al., 2019; Thompson & Higgins, 2002). Analyses used metafor (Viechtbauer, 2010) in R 4.5.2 with a fixed random seed. The independent re-extraction of the primary studies is described in Section 3.5. Effect magnitudes were interpreted using the L2-specific reference values *r* ≈ 0.25, 0.40, and 0.60 for small, medium, and large associations (Plonsky & Oswald, 2014).

## 4. Results

### 4.1. Study Characteristics

The eleven included studies (Table 1), published between 2009 and 2026, sampled 1250 L2 learners. The evidence base was geographically concentrated: ten of the eleven were conducted in Chinese-speaking settings—six on the Chinese mainland, three in Taiwan, and one in Hong Kong—and the eleventh sampled Egyptian undergraduates. Nine studies taught English as the target language; two taught Chinese—Peng et al. (2020), Mandarin pronunciation, and Puri et al. (2025), Chinese to international students in China. Those international students, together with the Egyptian undergraduates sampled by Mekheimer (2026), are the only non-Chinese-background samples in the pool. The studies covered writing (*k* = 3), interpreting (*k* = 2), listening (*k* = 2), speaking or pronunciation (*k* = 2), vocabulary (*k* = 1), and general classroom learning (*k* = 1). Ten measured cognitive load subjectively, most often through mental-effort ratings, a differentiated load scale, or a single-item difficulty rating; one used mean dependency distance as an objective processing-load index. Anxiety was assessed with skill-specific instruments or the Foreign Language Classroom Anxiety Scale, with one exception: Peng et al. (2020) used the anxiety subscale of the Achievement Emotions Questionnaire, an achievement-emotion rather than a language-specific instrument, and in ten of the eleven studies the instrument referred to the course or classroom as a whole rather than to the specific task being performed—a feature that bears directly on the exploratory pacing analysis reported in Section 4.3.

**Figure 2:**
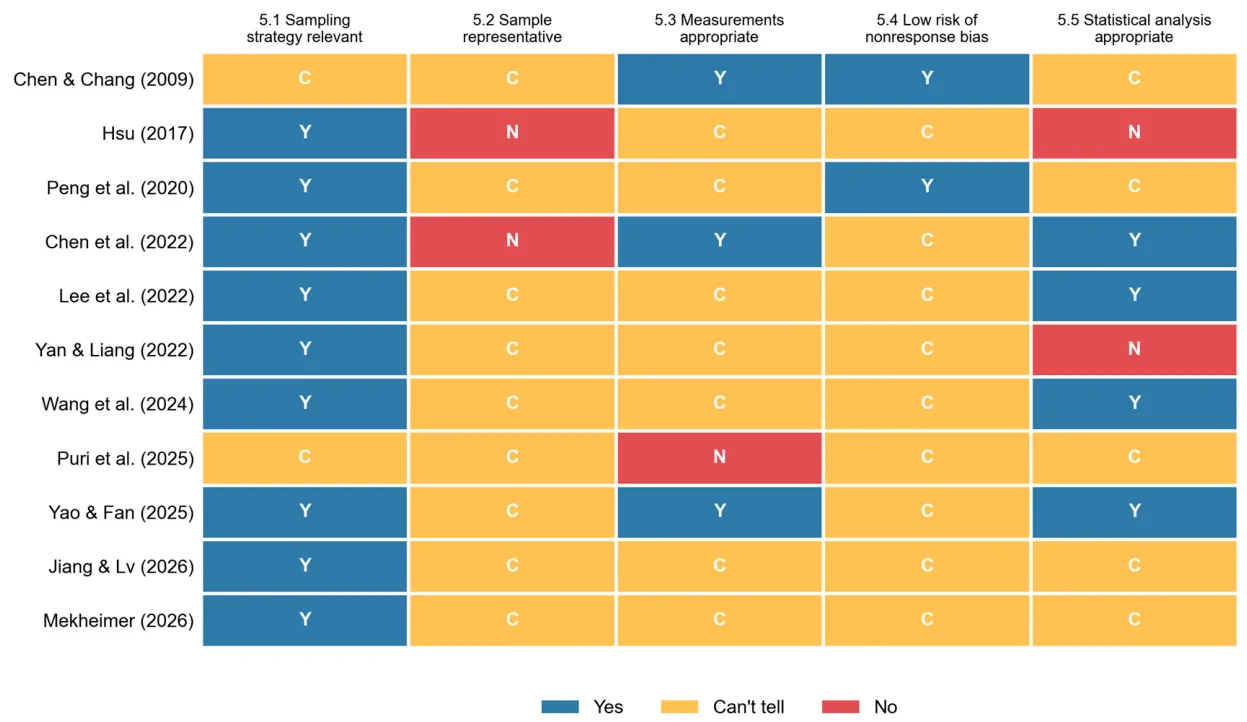
MMAT (Hong et al., 2018) criterion-level quality appraisal of the eleven included studies (Yes/Can’t tell/No). No summed score is computed.

The MMAT appraisal is reported at criterion level (Figure 2); no summed score is given, because the five criteria address different threats and are not commensurable. The profile varied substantially across criteria. Sampling strategy was judged relevant to the research question in nine of the eleven studies, whereas statistical analysis was judged appropriate in four and inappropriate in two. The weaknesses are concentrated in two criteria. Sample representativeness was rated Yes in none of the eleven studies: nine were rated Can’t tell, because single-institution convenience samples are described without a sampling frame, and two were rated No, because the sample was an intact class or a single cohort explicitly not intended to represent a wider population. Risk of nonresponse bias could not be judged in nine studies, which report neither response rates nor attrition. Measurement was judged appropriate in three studies, questionable in seven, and inadequate in one: Puri et al. (2025) report internal-consistency coefficients between 0.96 and 0.99 for short self-report scales, values that indicate item redundancy or undifferentiated responding rather than reliability. Two studies were rated No on statistical analysis: Yan and Liang (2022), whose reported correlation carries a sign that its own abstract contradicts, and Hsu (2017), whose subgroup correlations were not part of the stated research questions. A third study raises a concern that the MMAT criteria do not capture. Mekheimer (2026) was rated Can’t tell on statistical analysis, but its covariate correlation matrix contains values—including *r* = −0.951 between listening anxiety and comprehension accuracy at *N* = 60, obtained with a two-item anxiety scale—that are difficult to reconcile with the measures described. That study is the most influential in the pool, so the estimate without it is reported alongside the pooled value throughout. The consequences of both ratings for the pooled estimate are examined in Section 4.4.

### 4.2. Overall Association (Hypothesis 1)

**Figure 3:**
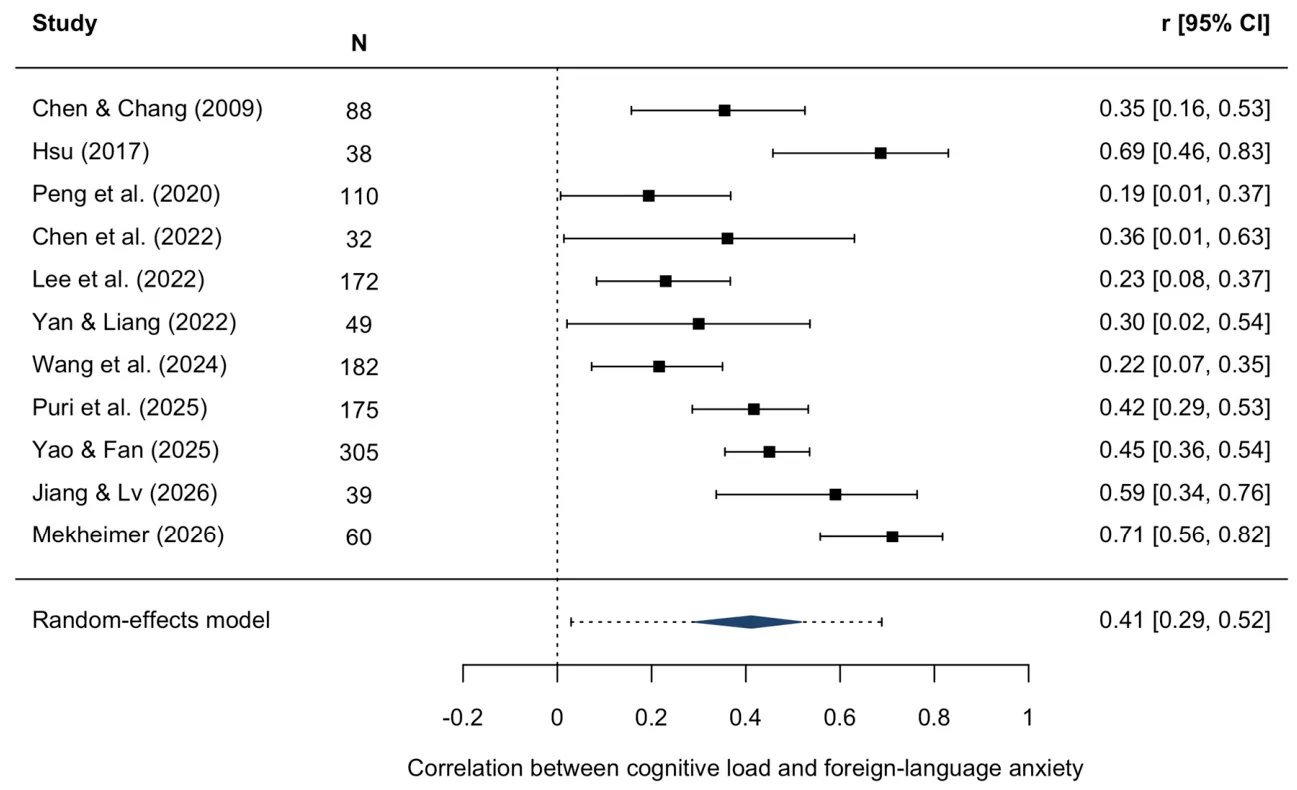
Forest plot of the cognitive load–anxiety correlation in L2 learning (random-effects, REML).

Across eleven studies, cognitive load and anxiety were positively associated, *r* = 0.41, 95% CI [0.29, 0.52], *z* = 6.33, *p* < .001 (Figure 3). All eleven study-level correlations were positive, ranging from 0.19 to 0.71. Relative to the L2-specific benchmarks of Plonsky and Oswald (2014), the pooled value sits between the medium and large reference points, but the interval is wide and the studies are few, so the point estimate must be interpreted alongside the heterogeneity reported next. Hypothesis 1 was supported.

### 4.3. Heterogeneity and the Exploratory Pacing Contrast

Between-study heterogeneity was substantial, *Q*(10) = 40.49, *p* < .001, *I*^2^ = 80.1%, *τ*^2^ = 0.039. The 95% prediction interval, [0.03, 0.69], is central to interpretation because it indicates that a future study drawn from the same underlying population could yield an association ranging from effectively zero to very strong. The pooled mean is therefore a statement about the average of a heterogeneous literature, not a value that individual studies can be expected to reproduce. Three features of the pool help account for the spread. The two largest coefficients come from two of the smallest samples—Hsu (2017), *r* = 0.71 at *N* = 38, and Mekheimer (2026), *r* = 0.71 at *N* = 60—although the pattern is not general: the smallest sample in the pool, Y. Chen et al. (2022) at *N* = 32, returned *r* = 0.36, and a meta-regression of Fisher’s z on sample size was not significant, QM(1) = 1.04, *p* = 0.31, explaining none of the heterogeneity. Second, the load indices are not equivalent operationalisations: they range from a single-item difficulty rating through differentiated multi-scale instruments to an objective syntactic index, and even within the three writing studies, which use the most similar instruments to one another, the estimates run from 0.22 and 0.23 to 0.45. Third, learner age and setting vary from Grade 3 children learning vocabulary through an augmented-reality game to postgraduate interpreting trainees. The exploratory pacing contrast was not significant, QM(1) = 0.11, *p* = .74, with real-time tasks at *r* = 0.43, 95% CI [0.27, 0.56], *k* = 7, and self-paced tasks at *r* = 0.39, 95% CI [0.17, 0.58], *k* = 4. Two features of the evidence prevent this analysis from supporting a substantive interpretation. First, pacing is not separable from language skill. Under the classification used in the original submission, in which the self-paced category contained exactly the three writing studies, the pacing contrast and a writing-versus-other-skills contrast returned numerically identical tests, QM(1) = 1.55, *p* = .21 in both cases, because they were the same partition of the data under two different names. Independent recoding assigned a fourth study, Hsu’s (2017) self-directed vocabulary game, to the self-paced category, which reduces the contrast to the non-significant value reported above; either way, the analysis cannot distinguish task pacing from language skill. Second, the anxiety instruments are largely at the wrong temporal grain for the question: in ten of the eleven studies, the instrument refers to the course or classroom rather than to the paced task itself, so the classification describes the task while the measurement describes the semester. The contrast is accordingly reported as exploratory and confounded, and no interpretive weight is placed on the numerical difference between the two subgroups. The load modality contrast could not be evaluated, since only one study used an objective index.

### 4.4. Small-Study Effects, Selective Non-Reporting, and Sensitivity Analyses

**Figure 4:**
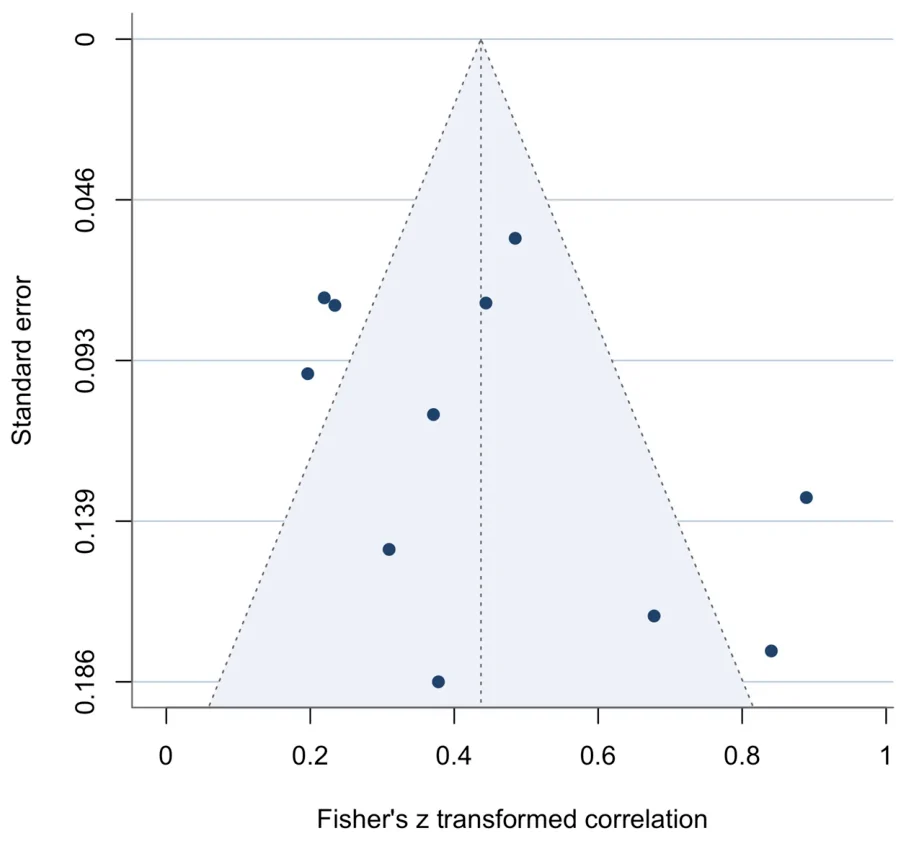
Funnel plot of the eleven included studies (Fisher’s *z*).

The analyses distinguish two threats because conventional diagnostics address only the first. The first is study-level publication or small-study bias among the eleven reports that did provide a coefficient. The funnel plot was broadly symmetric (Figure 4); neither the rank-correlation test, *τ* = 0.27, *p* = .28, nor Egger’s regression, *z* = 1.70, *p* = .09, reached significance, and trim and fill imputed no missing studies. At *k* = 11, these low-powered tests provide little reassurance. The second threat is selective non-reporting within studies that measured both constructs, and it is the more immediate problem in this dataset. Thirteen of the 29 reports (two of them judged from bibliographic records and abstracts alone, because their full texts could not be obtained) carried forward measured cognitive load and anxiety in the same learners yet published no statistic linking them. If authors are more likely to report that association when it is significant, positive, or theoretically convenient, the eleven available coefficients are a biassed sample of the coefficients that were computed, and the pooled estimate is correspondingly too high. A symmetric funnel plot among the eleven cannot speak to the thirteen that are absent, and neither can Egger’s test, the rank-correlation test, or trim and fill, all of which operate on the effects that were reported. We did not contact the authors of those thirteen reports to request the missing coefficients; this threat is therefore neither quantified nor corrected, and it is stated as the principal limitation of the pooled estimate in Section 5.6. Sensitivity analyses addressed the analytic decisions instead. Leave-one-out estimates ranged from *r* = 0.37 to *r* = 0.43. Removing Mekheimer (2026)—the study added during independent re-screening, and the one whose reported correlation matrix is hardest to reconcile with its stated measures—gave *r* = 0.37, 95% CI [0.27, 0.46], with *I*^2^ falling to 66.4%; this was the most influential leave-one-out result, and both estimates are reported wherever the pooled value is discussed. Restricting the pool to zero-order Pearson coefficients gave *r* = 0.43, *k* = 9; removing the one objective load study gave *r* = 0.42, *k* = 10; disaggregating the subgroup study gave *r* = 0.42 across 12 samples; and adding back the study excluded for pairing an objective load index with a general anxiety inventory gave *r* = 0.48, *k* = 12, with *I*^2^ rising to 91.5%. A full specification analysis addressed the one-effect-per-study rules. Substituting, for each study in turn, every alternative load component, measurement wave, subscale, subgroup, or path that the source reported—including, for the one longitudinal study, all fourteen coefficients in its correlation table that were not used in the primary model, among them the four the source reports as non-significant—moved the pooled estimate between *r* = 0.38 and *r* = 0.45. Two further specifications set every study simultaneously to its least and to its most favourable alternative; these bound the estimate at *r* = 0.29, 95% CI [0.15, 0.42], and *r* = 0.52, 95% CI [0.38, 0.64]. The most adverse single substitution, reversing the sign of the disputed dependency-distance coefficient discussed in Section 5.6, gave *r* = 0.38, 95% CI [0.20, 0.52]. No specification in this analysis produced a confidence interval containing zero.

## 5. Discussion

The central finding is straightforward: learners who experienced greater cognitive load also tended to report greater foreign-language anxiety. Across eleven independent samples (*N* = 1250), the pooled correlation was *r* = 0.41, 95% CI [0.29, 0.52], and *r* = 0.37, 95% CI [0.27, 0.46], when the one study whose reported correlation matrix could not be reconciled with its stated measures was removed. All eleven study-level estimates were positive, although their magnitudes varied substantially and the prediction interval extends almost to zero. The synthesis therefore documents positive covariation across the available estimates, a pattern relevant to working-memory constraints and attentional control, but it does not identify the process that produced that pattern.

### 5.1. Interpreting the Association (Hypothesis 1)

The size of the association is informative. A pooled r of 0.41—or 0.37 with the most influential study removed—indicates a meaningful relationship, but not an identity: cognitive load and language anxiety are connected yet remain distinct constructs. Positive estimates appeared in writing, speaking, interpreting, listening, vocabulary, and general classroom contexts, suggesting that the pattern is not confined to one language skill in the current evidence base. The result is consistent with MacIntyre and Gardner’s (1994) account of a cognitive component to language anxiety. It does not, however, test a mechanism. Executive-resource competition, appraisals of low control, proficiency, task difficulty, shared measurement method, or a combination of these factors could all produce the observed relationship.

Attentional control and control-value accounts together motivate a reciprocal hypothesis: demanding tasks may elicit anxiety, while anxiety-related worry may further reduce processing efficiency. Such feedback could make a task feel progressively more effortful. The concurrent evidence does not test such a feedback loop. They show only that learners who reported or exhibited greater load also tended to report greater anxiety. No included study manipulated anxiety to test whether reducing it subsequently lowers load.

### 5.2. The Exploratory Pacing Contrast and the Modality Question

The exploratory pacing contrast provided no evidence that the association differs by task pacing, QM(1) = 0.11, *p* = .74, and the analysis cannot support an inference either way. As Section 4.3 sets out, the classification is confounded with language skill—under the original coding it was arithmetically identical to a writing-versus-other contrast—and the anxiety instruments in ten of the eleven studies refer to the course rather than to the paced task. The finding is therefore that the question remains open, not that pacing does not matter. Nor would a significant pacing effect have distinguished attentional control from control-value explanations, because real-time tasks can simultaneously increase executive demand and reduce perceived control. Testing the boundary condition properly requires primary studies that measure anxiety at the level of the task whose pacing is manipulated, and that vary pacing within a single skill so that the two are not confounded by design. The exploratory measurement modality question was likewise not evaluable: only one pooled study measured load objectively, and a single observation cannot establish a modality difference. Primary studies that pair objective load measures with language-specific anxiety are needed.

### 5.3. Relation to Prior Work

The present result and the adjacent synthesis by Gullo et al. (2025) address different constructs. Their negative pooled association between foreign-language anxiety and an omnibus category of cognition combined working memory, attention, need for cognition, and cognitive load. Because greater capacity and greater load have opposite functional meanings, the sign of an aggregate effect depends on which components dominate. The current positive estimate for load is therefore not contradictory; it supplies a construct-specific value that the omnibus category cannot provide. The present review also reports heterogeneity and a prediction interval and includes an independent repetition of screening and extraction, thereby presenting the estimate together with its principal uncertainties. Table 2 summarises the comparison.

The magnitude also merits comparison with the wider literature. Foreign-language anxiety correlates negatively with achievement at approximately *r* = −0.36 (Teimouri et al., 2019) and *r* = −0.30 (X. Zhang, 2019; see also Botes et al., 2020), while working-memory capacity correlates with creative output at *r* = 0.08 across 28 samples (Gong et al., 2023). The comparisons are descriptive, not commensurate. The present estimate is based mainly on two self-report measures administered in the same session; the achievement estimates link self-report to performance, and the working-memory estimate links performance measures. Mono-method correlations are vulnerable to shared response and state effects. Even with that qualification, *r* = 0.41 places load among the potentially consequential cognitive correlates of foreign-language anxiety and motivates direct tests of whether processing demand helps explain anxiety’s relation to achievement.

### 5.4. Theoretical and Practical Implications

For intelligence research, the pooled estimate supplies a specific empirical target for models of L2 performance. It also clarifies which constructs should remain separate: working-memory capacity describes available cognitive resources, experienced load describes the demands placed on those resources, attentional control concerns how they are directed, and anxiety describes an affective state that may both influence and respond to processing. The findings are consistent with an intersection between load and anxiety at the level of limited processing resources, but they do not show that either construct causes the other. Future models should therefore compare common-cause, directional, and reciprocal explanations.

The practical lesson is not that every anxiety-reducing feature will also reduce cognitive load. It is that both outcomes should be measured when pacing, scaffolding, worked examples, segmented input, captions, on-demand support, or interface complexity are evaluated (Mayer, 2009; Moreno & Mayer, 2007). Pre-task planning and retrieval practice may free working-memory resources, but any affective benefit should be measured rather than assumed. Mobile, augmented-reality, and agent-based studies have reported lower load and lower anxiety together (Y. Chen et al., 2022; Peng et al., 2020; Puri et al., 2025), yet none establishes which change came first. Z. Wang and Pang (2026) further showed why an affective design feature cannot be assumed to be cognitively benign: they found no overall advantage of an emotional over a regular AI agent and reported exploratory evidence that verbose emotional scaffolding may add processing demands during challenging L2 vocabulary learning, particularly for learners with lower baseline proficiency. Because those subgroup and load-performance patterns were exploratory and partly correlational, they identify a possible boundary condition rather than a mechanism. The present synthesis therefore supports measuring cognitive and affective outcomes together; it does not validate a particular instructional technique.

### 5.5. A Reporting Gap in the Primary Literature

The screening record identifies a consequential reporting problem, and the independent re-screening reported in Section 3.5 showed that it is compounded by where such statistics are placed. Among the 29 reports carried forward for detailed construct-level disposition, eleven reported a poolable linking statistic and thirteen measured both constructs but provided none; four measured only one construct, or treated load as a manipulation rather than a measured score, on full-text inspection, and one paired an objective load index with a general rather than a language-specific anxiety measure. More than half of the reports that measured both constructs therefore left the within-sample relationship unavailable for synthesis. One further case is instructive. Mekheimer (2026) does report the bivariate correlations, but places them in a table of covariate checks introduced as support for including two covariates, while the analysis of cognitive load in the results proper is a mixed model in which anxiety appears only as an adjusted term. The usable statistic was identified only during the independent full-text re-screening. Four reporting practices would improve the evidence base. First, studies administering both a load index and an anxiety scale should report their bivariate association regardless of the focal hypothesis. Second, that association should be reported in the results rather than embedded in a preliminary or covariate table. Third, load should be reported by component where the instrument distinguishes intrinsic, extraneous, and germane processing. Fourth, anxiety measurement should match the skill and temporal level of the task. These practices would make existing data more informative without adding participant burden.

### 5.6. Limitations and Future Directions

Six limitations constrain interpretation. The first three concern the evidence base and the statistical synthesis. First, and most seriously, selective non-reporting of the focal association is not correctable with the data available. Thirteen of the 29 reports (two of them judged from bibliographic records and abstracts alone, because their full texts could not be obtained) carried forward measured both constructs and published no statistic linking them, and we did not approach their authors for the missing coefficients. If those coefficients were withheld more often when they were small or inconvenient, the eleven that were published are a biassed sample and *r* = 0.41 is too high. This is a different problem from publication bias, and the funnel plot, Egger’s test, the rank-correlation test, and trim and fill—all of which analyse the effects that were reported—cannot address it. It is the principal limitation of the synthesis; the pooled value is therefore best regarded as an upper bound rather than an unbiased estimate. Second, the synthesis contains eleven independent samples. This number is sufficient to estimate a random-effects mean but affords limited power for moderators and small-study-effect tests (Jackson & Turner, 2017; Valentine et al., 2010). Meta-analyses with similarly small pools have appeared across psychological journals: *k* = 5 in *Behavioural and Cognitive Psychotherapy* (Wojnarowski et al., 2019), *k* = 9 in *Child and Adolescent Mental Health* (Fulambarkar et al., 2022), *k* = 11 in the *Journal of Child Psychology and Psychiatry* (Abramovitch et al., 2015), and *k* = 10 in *Journal of Intelligence* (Te Nijenhuis & Van den Hoek, 2016). These precedents show that an eleven-study synthesis is not unprecedented, but they do not remove its uncertainty. Third, heterogeneity was high, *I*^2^ = 80.1%, and the prediction interval, [0.03, 0.69], reaches almost zero. The pooled mean describes the centre of a dispersed literature; it is not a value that a new study can be expected to reproduce. That interval is itself uncertain, because between-study variance was estimated from eleven studies, and it should be read as a model-based description of dispersion rather than as a calibrated forecast.

The remaining three limitations concern measurement and the review process. Fourth, common-method variance is a major alternative explanation: ten of the eleven studies measured both constructs by self-report, usually in one session, so the subjective-load estimate does not provide method-independent confirmation. The single objective load study is also the least secure input in the pool. Yan and Liang (2022) print a positive correlation between interpretation-classroom anxiety and mean dependency distance in their Table 5, while their abstract describes the same association as negative, and they map shorter dependency distance onto higher cognitive load, inverting the usual convention in which longer dependencies index greater processing difficulty. Under either internally consistent reading, the anxiety–load association is positive at about 0.30, which is the value synthesised here; taking the printed sign together with the authors’ inverted mapping would reverse it, and that substitution is reported in Section 4.4. Fifth, proficiency and learner age are plausible common causes that could not be modelled. Less proficient learners may experience both greater processing demand and greater language anxiety, producing a positive correlation without direct resource competition, and the pool spans Grade 3 children to postgraduate interpreting trainees; neither variable was reported comparably enough for moderator coding. Related to this, the evidence base is geographically narrow. Ten of the eleven studies were conducted in Chinese-speaking settings and nine taught English, so the estimate should not be read as a general statement about L2 learners. Whether the association differs where English is not the target language, where instruction is less examination-oriented, or where classroom norms around error and evaluation differ, is unknown, and replication outside these settings is a priority for future research. Finally, the review process itself has two weaknesses. The review was not prospectively registered, and no protocol was deposited in advance; the eligibility criteria and the pacing contrast were fixed before the corresponding models were fitted, and the full screening record, extraction sheet, and analysis scripts are released so that every decision can be audited after the fact, but an unregistered review cannot demonstrate to a reader that its decisions were settled beforehand. The audit trail is also incomplete for the supplementary sweep: eight reports excluded at full text were not individually logged, so they cannot be listed with reasons as PRISMA 2020 requires. In addition, although the full-text stage was re-done independently by two machine coders and verified against the sources by two of the authors, the title-and-abstract screening of 100 records was performed once, by the first author; a study wrongly excluded at that stage would not have been recovered by the later independent check.

Three research designs would be particularly informative. Cross-lagged panel or intensive longitudinal studies could test whether load predicts later anxiety beyond prior anxiety and whether anxiety predicts later load beyond prior load. Experiments could manipulate intrinsic and extraneous load independently—through element interactivity and presentation format—while measuring both perceived load and objective processing indicators. Cross-cultural and cross-skill replications beyond the predominantly Chinese-speaking, writing- and interpreting-heavy evidence base would test generalisability. Registered reports would strengthen all three designs and reduce the selective reporting that made many otherwise relevant studies unusable.

## 6. Conclusions

In plain terms, learners who experienced greater cognitive load also tended to experience greater foreign-language anxiety. Across eleven studies (*N* = 1250), the pooled association was *r* = 0.41, 95% CI [0.29, 0.52], and *r* = 0.37, 95% CI [0.27, 0.46], with the most influential study removed. The relationship varied considerably across studies: its model-based 95% prediction interval, [0.03, 0.69], remains positive but reaches almost zero, so the pooled value should be read as the centre of a dispersed literature rather than as an expected result for any new study. The review also showed how often this relationship has been left unavailable: only eleven of the 29 reports carried forward for detailed construct-level assessment reported a statistic linking the constructs, and one of those statistics was identified only through independent full-text re-screening.

The pooled estimate was relatively stable across the sensitivity analyses conducted. All eleven study-level correlations were positive; leave-one-out estimates ranged from 0.37 to 0.43; a specification analysis that substituted every available alternative load component, measurement wave, subscale, subgroup, and path yielded estimates from 0.38 to 0.45; and setting every study simultaneously to its least and most favourable alternative widened the range to 0.29–0.52. None of these specifications produced an interval containing zero. Stability across the checks that were run is not the same as robustness to all reasonable analytic choices, and it says nothing about the coefficients that were never reported. The exploratory pacing contrast was not significant, and because task pacing is confounded with language skill in this pool and the anxiety instruments largely refer to the course rather than the task, it should be treated as a question still to be tested rather than as a null result. With only one objective load study, measurement modalities could not be compared at all.

The contribution to intelligence research is a clearer connection between cognitive architecture and affect in L2 performance. Experienced processing demand and language-related anxiety should be examined jointly within models of working memory and attentional control, while remaining conceptually distinct. The positive association is consistent with limited-resource accounts, but the concurrent, predominantly self-report evidence cannot distinguish executive competition from low-control appraisal, proficiency, task difficulty, or shared method variance.

The next step is therefore to establish sequence and mechanism. Studies should measure load and anxiety over time, manipulate processing demand and perceived control separately, and combine subjective with objective indicators. Such designs can test whether the relationship is directional, reciprocal, or produced by common causes, and whether it generalises across languages, skills, cultures, and levels of cognitive ability. This meta-analysis supplies the quantitative starting point for those tests, not their causal conclusion.

## Figures and Tables

**Table 1:** Characteristics and effect sizes of the eleven included studies.

Study	Setting	*N*	Skill	Anxiety Instrument (Level)	Cognitive Load Index (Modality)	Pacing	Source Statistic	*r*
I.-J. Chen and Chang (2009)	Taiwan, China	88	Listening	FLCAS, 33 items (course)	CLSRS mental effort, 7-point (subj.)	Real-time	Zero-order Pearson *r*	0.35
Hsu (2017)	Taiwan, China	38	Vocabulary	FLCAS adapted, 8 items (course)	Mental-effort items, 3 of 8 CLQ items (subj.)	Self-paced	Two subgroup r, weighted Fisher z	0.69
Peng et al. (2020)	Mainland China	110	Speaking	AEQ anxiety, during learning (task)	Intrinsic load, single item (subj.)	Real-time	Zero-order Pearson *r*	0.19
Y. Chen et al. (2022)	Mainland China	32	General	FLCAS, 28 items (course)	Intrinsic load subscale (10-item CLS) (subj.)	Real-time	Spearman’s rho, final wave	0.36
Lee et al. (2022)	Taiwan, China	172	Writing	SLWAI, 22 items (course)	Content load, single item (subj.)	Self-paced	Zero-order Pearson *r*	0.23
Yan and Liang (2022)	Hong Kong, China	49	Interpreting	ICFLA, 15 items (course)	Mean dependency distance (obj.)	Real-time	Zero-order Pearson *r*	0.30
H. Wang et al. (2024)	Mainland China	182	Writing	Short L2 writing anxiety, 9 items (course)	Writing load subscale, 5 items (subj.)	Self-paced	Zero-order Pearson *r*	0.22
Puri et al. (2025)	Mainland China	175	Speaking	FLSAS, 13 items; LCLA, 4 items (course)	Leppink load total, 10 items (subj.)	Real-time	Two bivariate betas (=*r*), Fisher z	0.42
Yao and Fan (2025)	Mainland China	305	Writing	SLWAI, 22 items (course)	CL-AI-L2W total, 18 items (subj.)	Self-paced	Zero-order Pearson *r*	0.45
Z. Jiang and Lv (2026)	Mainland China	39	Interpreting	Interpreting Anxiety Scale, 20 items (course)	Perceived task difficulty, 1 item (subj.)	Real-time	Zero-order Pearson *r*	0.59
Mekheimer (2026)	Egypt	60	Listening	Listening anxiety, 2 items (course)	Perceived load, 3 items (subj.)	Real-time	Two within-participant *r*, Fisher *z*	0.71

Note. Setting follows the location of data collection. Anxiety temporal level distinguishes instruments that refer to a specific task from those that refer to the course or classroom as a whole. Load modality distinguishes subjective self-report from objective behavioural measurement. Task pacing is the exploratory moderator defined in Section 2.4. The source statistic column reports how the synthesised coefficient was obtained; the underlying values and every alternative that was considered appear in Supplementary File S1.

**Table 2:** Comparison with the most closely related synthesis.

Feature	Gullo et al. (2025)	Present Study
Focal cognitive construct	Omnibus “cognition” (load, working memory, attention, need for cognition)	Cognitive load only
Number of studies (*k*)	13	11
Anchoring of the relationship	Anxiety vs. cognitive performance/cost	Load–anxiety concurrent covariation
Pooled effect	*θ* = −0.28	*r* = 0.41 (*r* = 0.37 excluding the most influential study)
Heterogeneity and dispersion	Not reported in comparable form	*I*^2^ = 80.1%; 95% prediction interval [0.03, 0.69]
Independent verification of screening and extraction	Not reported	Full-text stage repeated by two independent machine coders and verified against the sources by two of the authors (*κ* = 1.00 for inclusion)
Moderation by load modality/task pacing	Not possible (constructs merged)	Pacing contrast exploratory and confounded with skill (*k* = 7 vs. 4, ns); load modality not evaluable (*k* = 1 objective study)

## Data Availability

All data supporting the reported results are provided as Supplementary Material, and no separate repository deposit was made. All included studies are published and are cited in the reference list.

## Supplementary Materials

The supporting information can be downloaded at https://www.mdpi.com/article/10.3390/jintelligence14090228/s1. Supplementary File S1 contains the full database search strategies, the twenty supplementary Crossref query combinations, the coding manual given to the two machine coders, their record-level codings, the agreement statistics between them, and the documented resolution of every discrepancy. It also lists, for each of the 29 reports carried forward for construct-level disposition, the disposition assigned to it: the 11 that entered the primary synthesis and the 18 that did not. Of those 18, 13 measured both constructs without reporting a poolable linking statistic, four measured only one construct or treated load as a manipulation, and one paired an objective load index with a general anxiety inventory. The single retracted record was identified and excluded at title-and-abstract screening and is therefore not among the 29. Supplementary File S2 is the completed PRISMA 2020 checklist. Supplementary File S3 contains the coded extraction sheet, the effect-size table used in every analysis, the screening counts, the criterion-level MMAT appraisal, and a codebook. Supplementary File S4 contains the R/metafor analysis scripts.
