# Supplementary material for: Cognitive Load and Foreign-Language Anxiety in Second-Language Learning: A Systematic Review and Meta-Analysis
Source: J Intell. 2026 Sep 21;14(9):228. doi: 10.3390/jintelligence14090228 (PMC13608070; doi:10.3390/jintelligence14090228)
Supplement: Supplementary file 1 [file jintelligence-14-00228-s001.zip › SupplementaryFileS2_PRISMA2020_checklist_R1.pdf]

PRISMA 2020 Checklist

Cognitive Load and Foreign-Language Anxiety in Second-Language Learning: A Meta-Analysis

Manuscript ID jintelligence-4520750 · Journal of Intelligence

Item wording follows Page et al. (2021), BMJ 372, n71. Locations refer to the first revision (R1, 5 September 2026), in which the full-text stage was independently double-coded, the pool changed from ten to eleven studies, and the intervention analysis was removed. Items the review did not undertake are marked as such.

Section and Topic	Item #	Checklist item	Location where item is reported
**TITLE**			
Title	1	Identify the report as a systematic review.	Title. Identified as a meta-analysis; the systematic search and screening procedure are reported in Section 3 and Figure 1.
**ABSTRACT**			
Abstract	2	See the PRISMA 2020 for Abstracts checklist.	Abstract. Reports objective, eligibility, sources, synthesis method, k and N, the pooled estimate with its confidence interval, the estimate with the most influential study removed, heterogeneity and the 95% prediction interval, the independent double coding and its agreement, the exploratory status of the pacing contrast, the selective-non-reporting limitation, and the geographical restriction of the evidence base.
**INTRODUCTION**			
Rationale	3	Describe the rationale for the review in the context of existing knowledge.	Section 1, paragraphs 1–5.
Objectives	4	Provide an explicit statement of the objective(s) or question(s) the review addresses.	Section 1, final paragraph; Section 2.5. Hypothesis 1 is stated in Section 2.3. The pacing contrast is posed in Section 2.4 as an exploratory question rather than as a hypothesis, with the reasons stated there.
**METHODS**			
Eligibility criteria	5	Specify the inclusion and exclusion criteria for the review and how studies were grouped for the syntheses.	Section 3.2. Grouping for the synthesis is specified in Section 3.6.
Information sources	6	Specify all databases, registers, websites, organisations, reference lists and other sources searched or consulted to identify studies. Specify the date when each source was last searched or consulted.	Section 3.3 (Web of Science Core Collection, Scopus, EBSCOhost: ERIC, Academic Search Complete, PsycINFO, LISTA) and Section 3.4 (supplementary bibliographic sweep). Search dates are given in Section 3.3.
Search strategy	7	Present the full search strategies for all databases, registers and websites, including any filters and limits used.	Section 3.3 gives the Web of Science strategy verbatim and states that Scopus and EBSCOhost used the same Boolean logic with platform-specific field tags. The full verbatim strategy for each platform, together with the twenty supplementary query combinations, is provided in Supplementary File S1. No date, language, or document-type filters were applied.
Selection process	8	Specify the methods used to decide whether a study met the inclusion criteria of the review, including how many reviewers screened each record and each report retrieved, whether they worked independently, and if applicable, details of automation tools used in the process.	Sections 3.1, 3.4, and 3.5. Title-and-abstract screening of the 100 unique records was carried out by the first author alone. All 29 full-text eligibility decisions were then re-made independently by Authors A and B working blind to the first author's decisions and to each other; agreement on primary-synthesis inclusion was complete ($\kappa = 1.00$), agreement with the first author's original disposition was $\kappa = 0.854$, and all discrepancies were resolved against the source reports by Author C and documented in Supplementary File S1. The single-screener title-and-abstract stage is reported as a limitation in Section 5.6. Automation was limited to de-duplication on normalised DOI and title.
Data collection process	9	Specify the methods used to collect data from reports, including how many reviewers collected data from each report, whether they worked	Section 3.5. Extraction was carried out by the first author and then repeated independently by Authors A and B for every included study,

		independently, any processes for obtaining or confirming data from study investigators, and if applicable, details of automation tools used in the process.	working from the source reports and a written coding manual. Two extraction or classification errors were identified and corrected in this way; both are described in Section 3.5 and itemised in Supplementary File S1.
Data items	10a	List and define all outcomes for which data were sought. Specify whether all results that were compatible with each outcome domain in each study were sought, and if not, the methods used to decide which results to collect.	Sections 3.5 and 3.6. The outcome is the association between cognitive load and foreign-language anxiety. Exactly one estimate was retained per study under the rule stated in Section 3.5 (the study's most inclusive index of load, falling back on the intrinsic component only where components are reported without a total; the final wave where measurements were repeated). All alternatives reported by the source studies were retained and are analysed in the specification analysis in Section 4.4.
Data items	10b	List and define all other variables for which data were sought (e.g. participant and intervention characteristics, funding sources). Describe any assumptions made about any missing or unclear information.	Section 3.5: sample size, setting, target language and skill, anxiety instrument with its temporal level, cognitive-load instrument with its modality, task pacing, and the source statistic. All of these are reported study by study in Table 1. Section 3.6 describes each non-single-Pearson input in full.
Study risk of bias assessment	11	Specify the methods used to assess risk of bias in the included studies, including details of the tool(s) used, how many reviewers assessed each study and whether they worked independently, and if applicable, details of automation tools used in the process.	Section 3.5. Mixed Methods Appraisal Tool (2018 version; Hong et al., 2018), five quantitative-descriptive criteria. Each criterion was rated by the first author and independently by Authors A and B; agreement across the 55 study-by-criterion cells was 85.7%. Following MMAT guidance, ratings are reported at criterion level and no summed quality score is computed.
Effect measures	12	Specify for each outcome the effect measure(s) (e.g. risk ratio, mean difference) used in the synthesis or presentation of results.	Section 3.6: Fisher's z for pooling, back-transformed to r for reporting. No other effect-size metric is used; the intervention analysis present in the previous version has been removed.
Synthesis methods	13a	Describe the processes used to decide which studies were eligible for each synthesis.	Sections 3.2 and 3.6; the disposition of every full-text report is shown in Figure 1 and listed in Supplementary File S1.
Synthesis methods	13b	Describe any methods required to prepare the data for presentation or synthesis, such as handling of missing summary statistics, or data conversions.	Section 3.6, which states for each study how the synthesised coefficient was obtained: two simple bivariate standardised paths that are themselves zero-order correlations and were averaged on the Fisher-z scale; one Spearman coefficient; two inverse-variance-weighted subgroup Pearson correlations; and two within-participant condition correlations averaged on the Fisher-z scale.
Synthesis methods	13c	Describe any methods used to tabulate or visually display results of individual studies and syntheses.	Table 1 (study-level characteristics and correlations), Figure 3 (forest plot), Figure 4 (funnel plot), Figure 2 (MMAT appraisal).
Synthesis methods	13d	Describe any methods used to synthesize results and provide a rationale for the choice(s). If meta-analysis was performed, describe the model(s), method(s) to identify the presence and extent of statistical heterogeneity, and software package(s) used.	Section 3.6: random-effects model estimated by restricted maximum likelihood, specified a priori; Cochran's Q, I-squared, tau-squared, and a 95% prediction interval; metafor 4.8.0 in R 4.5.2 with a fixed seed, independently reproduced in a separate Python implementation. Only one synthesis is reported; the intervention analysis present in the previous version has been removed, and the reasons are given in the response to reviewers.
Synthesis methods	13e	Describe any methods used to explore possible causes of heterogeneity among study results (e.g. subgroup analysis, meta-regression).	Section 3.6: mixed-effects meta-regression fitting one binary contrast at a time. The pacing contrast was fixed before model fitting but is reported as exploratory and confounded, for the reasons documented in Sections 2.4 and 4.3.
Synthesis methods	13f	Describe any sensitivity analyses conducted to assess robustness of the synthesized results.	Section 3.6 and Section 4.4: leave-one-out analysis; models removing the most influential study, the

			objective-load study, and the study rated inadequate on statistical analysis; a model adding back the reserved construct-mismatch study; disaggregation of the subgroup study; a zero-order-only model; and a full specification analysis substituting every alternative component, wave, subscale, subgroup, and path reported by the source studies.
Reporting bias assessment	14	Describe any methods used to assess risk of bias due to missing results in a synthesis (arising from reporting biases).	Section 3.6: funnel plot, Egger's regression, rank-correlation test, and trim and fill, interpreted as uninformative rather than reassuring at k = 11. Section 4.4 additionally treats selective non-reporting within studies that measured both constructs as a separate threat that these diagnostics cannot address.
Certainty assessment	15	Describe any methods used to assess certainty (or confidence) in the body of evidence for an outcome.	Not undertaken. No GRADE or comparable body-of-evidence certainty rating was applied. Methodological quality was appraised at the study level with the MMAT (Section 3.5, Figure 2), and the factors that would bear on certainty—the small number of studies, the reliance on self-report in nine of ten studies, and confounding by proficiency—are stated in Section 5.6.
**RESULTS**			
Study selection	16a	Describe the results of the search and selection process, from the number of records identified in the search to the number of studies included in the review, ideally using a flow diagram.	Section 3.4 and Figure 1 (PRISMA 2020 flow diagram, both arms).
Study selection	16b	Cite studies that might appear to meet the inclusion criteria, but which were excluded, and explain why they were excluded.	Section 3.4 and the Data Availability Statement give the counts and reasons for the 29 reports that reached construct-level assessment. PARTIAL: the eight reports excluded at full text from the supplementary Crossref sweep were not individually logged, so they cannot be cited with reasons. This departure is stated in Section 3.4 and in Section 5.6. For the 29: 11 entered the primary synthesis and 18 did not — 13 measured both constructs but reported no poolable linking statistic, four measured only one construct or treated load as an experimental manipulation, and one paired an objective load index with a general rather than a language-specific anxiety inventory. One retracted record was identified and excluded at title-and-abstract screening and never entered full-text assessment.
Study characteristics	17	Cite each included study and present its characteristics.	Section 4.1 and Table 1.
Risk of bias in studies	18	Present assessments of risk of bias for each included study.	Figure 2 (criterion level, no summed score) and Supplementary File S1, sheets S1d.
Results of individual studies	19	For all outcomes, present, for each study: (a) summary statistics for each group (where appropriate) and (b) an effect estimate and its precision (e.g. confidence/credible interval), ideally using structured tables or plots.	Table 1 and Figure 3.
Results of syntheses	20a	For each synthesis, briefly summarise the characteristics and risk of bias among contributing studies.	Section 4.1.
Results of syntheses	20b	Present results of all statistical syntheses conducted. If meta-analysis was done, present for each the summary estimate and its precision (e.g. confidence/credible interval) and measures of statistical heterogeneity. If comparing groups, describe the direction of the effect.	Sections 4.2 and 4.3.
Results of syntheses	20c	Present results of all investigations of possible causes of heterogeneity among study results.	Section 4.3. The pacing contrast is reported as exploratory and confounded with language skill; the load-modality contrast is reported as not evaluable at one objective-load study.
Results of syntheses	20d	Present results of all sensitivity analyses conducted	Section 4.4.

		to assess the robustness of the synthesized results.	
Reporting biases	21	Present assessments of risk of bias due to missing results (arising from reporting biases) for each synthesis assessed.	Section 4.4 and Figure 4.
Certainty of evidence	22	Present assessments of certainty (or confidence) in the body of evidence for each outcome assessed.	Not undertaken; see item 15. The corresponding uncertainties are discussed in Section 5.6.
**DISCUSSION**			
Discussion	23a	Provide a general interpretation of the results in the context of other evidence.	Sections 5.1, 5.2, and 5.3.
Discussion	23b	Discuss any limitations of the evidence included in the review.	Section 5.6, limitations one to four (selective non-reporting of the focal association; the small number of studies; high heterogeneity and a prediction interval reaching almost to zero; common-method variance and the internally inconsistent dependency-distance index).
Discussion	23c	Discuss any limitations of the review processes used.	Section 5.6, limitation six (absence of prospective registration and of a deposited protocol; single-screener title-and-abstract stage). The full-text stage was independently double-coded, as reported in Section 3.5.
Discussion	23d	Discuss implications of the results for practice, policy, and future research.	Sections 5.4 and 5.5, and the closing paragraph of Section 5.6.
**OTHER INFORMATION**			
Registration and protocol	24a	Provide registration information for the review, including register name and registration number, or state that the review was not registered.	Section 3.1. The review was not registered in PROSPERO or any other prospective register.
Registration and protocol	24b	Indicate where the review protocol can be accessed, or state that a protocol was not prepared.	Section 3.1. No protocol was prepared or published in advance.
Registration and protocol	24c	Describe and explain any amendments to information provided at registration or in the protocol.	Not applicable; there was no registration or protocol to amend.
Support	25	Describe sources of financial or non-financial support for the review, and the role of the funders or sponsors in the review.	Funding statement in the back matter: the research received no external funding.
Competing interests	26	Declare any competing interests of review authors.	Conflicts of Interest statement in the back matter.
Availability of data, code and other materials	27	Report which of the following are publicly available and where they can be found: template data collection forms; data extracted from included studies; data used for all analyses; analytic code; any other materials used in the review.	Data Availability Statement in the back matter. Supplementary File S1 contains the search strategies, coding manual, record-level codings by both independent coders (Authors A and B), agreement statistics, discrepancy resolutions, and the criterion-level MMAT appraisal. Supplementary File S3 contains the extraction sheet, the effect-size table used in every analysis, the screening counts, and a codebook. Supplementary File S4 contains the analysis and verification scripts.

Reference: Page, M. J., McKenzie, J. E., Bossuyt, P. M., Boutron, I., Hoffmann, T. C., Mulrow, C. D., et al. (2021). The PRISMA 2020 statement: An updated guideline for reporting systematic reviews. BMJ, 372, n71. https://doi.org/10.1136/bmj.n71
